# Protocol for an open-label feasibility study for a randomised controlled trial of vitamin D supplementation in Crohn’s Disease patients with vitamin D deficiency: D-CODE Feasiblity study

**DOI:** 10.1186/s40814-021-00813-3

**Published:** 2021-03-20

**Authors:** Jane Fletcher, Emma Bedson, Michaela Brown, Martin Hewison, Amelia Swift, Sheldon C. Cooper

**Affiliations:** 1 Queen Elizabeth Hospital Birmingham, University Hospitals Birmingham NHS Trust, Edgbaston, Birmingham, B15 2TH England; 2 Liverpool Clinical Trials Centre, University of Liverpool, 2nd Floor Institute in the Park, Alder Hey Children’s Hospital, Eaton Road, Liverpool, L12 2AP England; 3 Institute for Systems and Metabolism Research, University of Birmingham, Edgbaston, Birmingham, B15 2TT England; 4 School of Nursing, University of Birmingham, Edgbaston, Birmingham, B15 2TT England

**Keywords:** Crohn’s disease, Vitamin D, Deficiency, Supplementation, Screening, Cholecalciferol, Hepcidin, Patient-reported outcome measure

## Abstract

**Background:**

Crohn's disease (CD) is a principal form of inflammatory bowel disease, affecting approximately 1 in every 650 people in the UK. Vitamin D deficiency is common in approximately 57.7% of CD patients; with anaemia occurring in about 43% of patients. There is growing evidence that supplementing CD patients who are vitamin D deficient may be effective in reducing the severity of CD symptoms and reducing iron-deficiency anaemia. Nevertheless, National Institute for Health and Care Excellence guidance regarding the management of CD does not address vitamin D deficiency in these patients. The aims of the study are (1) to determine the prevalence of vitamin D deficiency in adults with CD in Birmingham, UK and (2) to assess the feasibility of conducting a multi-site randomised controlled trial in adult patients with CD and vitamin D deficiency.

**Methods:**

D-CODE consists of two parts—a screening study and an open-label randomised controlled feasibility study.

1. Vitamin D screening

Three hundred patients, 18 years or older with CD will have a dried blood spot test to measure vitamin D levels. Dietary and sun exposure data will be collected. Eligible patients with low levels of vitamin D will be invited to participate in the feasibility study.

2. Feasibility study

Fifty participants with CD and vitamin D deficiency will be randomised to receive either a low (400 IU daily for 24 weeks) or high (3200 IU daily for 12 weeks then vitamin D3 800 IU daily for 12 weeks) dose of vitamin D3 oral supplementation.

Patient-reported outcomes (Inflammatory Bowel Disease Questionnaire, EQ-5D-5L and Crohn’s Disease Activity Index Score) will be collected at weeks 0 and 24. Biochemical monitoring will take place at weeks 0, 12 and 24 and will measure 25-hydroxyvitamin D, corrected calcium, albumin, parathyroid hormone, hepcidin, other vitamin D metabolites, iron studies and C-reactive protein. Faecal calprotectin will be measured at weeks 0 and 24.

**Discussion:**

A key aspect of D-CODE is the identification of vitamin D deficiency prior to supplementation. It is hoped that this feasibility study will lead to a definitive trial that will investigate the benefits of treating vitamin D deficiency in patients with CD.

**Trial registration:**

The trial has been registered with EudraCT number 2018-003910-42, ClinicalTrials.gov identifier NCT03718182 and ISRCTN number 15717783.

## Introduction

### Background and rationale

Inflammatory bowel diseases (IBD) are both chronic and often debilitating [1]. IBD is characterised by a mixture of abdominal pain, diarrhoea, anaemia, fatigue and nutritional problems. Crohn's disease (CD) is one of the principal IBD diseases, affecting approximately 1 in every 650 people in the UK [1]. CD may affect any part of the gastrointestinal tract, from the mouth to the anus, causing inflammation and ulceration. Patients with active CD often report having a poor quality of life as a result [2]. CD diagnosis is made by a gastroenterologist in keeping with best clinical practice including radiology, endoscopy and histology where possible [3]. Confirmed diagnosis is recorded in the patient’s clinical documentation. Vitamin D deficiency is common in patients with CD [4, 5]. A recent meta-analysis of observational studies found that the overall prevalence of vitamin D deficiency in CD was 57.7% [6].

The cause of vitamin D deficiency in patients with CD is likely to be multifactorial with influencing factors including:

- Reduced sunlight exposure due to their disease-restricting activity [7]
- Recommendations from medical teams to avoid sunlight exposure, or use high protection sunscreen, due to the increased risk of skin cancer with immunosuppressive treatments such as thiopurines [8]
- Reduced dietary intake of vitamin D due to disease symptoms and activity [7]
- Malabsorption of fat-soluble vitamins such as vitamin D. This may be due to bile salt malabsorption, particularly associated with CD affecting the terminal ileum [9].

A role for vitamin D in iron homeostasis has been proposed [10] [10]. Vitamin D deficiency has been linked to anaemia in various clinical settings [11], notably in patients with chronic kidney disease [12]. The precise mechanism for this has yet to be fully defined but appears to be linked to direct regulation of the iron-regulatory protein hepcidin by the active form of vitamin D, 1,25(OH)2D [13]. Hepcidin acts by inhibiting cellular export of iron [14]. In the setting of inflammatory disorders such as Crohn’s disease, hepcidin levels are increased in enterocytes and immune cells, resulting in decreased circulating levels of iron [15]. Vitamin D appears to counteract this mechanism by transcriptionally suppressing hepcidin as part of a mechanism for inhibiting access to iron by intracellular pathogens [13].

The National Osteoporosis Society recommends vitamin D supplementation at blood levels of 25(OH) D of < 50 nmol/L in people who have conditions associated with malabsorption such as CD [16]. However, current National Institute for Health Excellence (NICE) guidance regarding the management of CD does not address vitamin D deficiency in these patients [17]. In this patient group, standardised vitamin D supplementation should be considered with regards to maintaining healthy bones in those receiving corticosteroids [18]. However, there is growing evidence that supplementing CD patients who are vitamin D deficient may be effective in both reducing the severity of CD symptoms, such as anaemia [13] and pain, and reducing the need for surgical intervention [19–21].

Nevertheless, determining an effective dose of vitamin D supplementation in patients with malabsorption is difficult. A recent review suggested that vitamin D doses ranging from 1800 to 10,000 IU daily may be necessary in patients with IBD [22].

Currently, there is no UK standard practice in terms of checking vitamin D levels or standard of care in terms of dose or route of supplementation for patients with CD. There is a lack of clear national guidance regarding routine screening and the management of vitamin D deficiency in patients with CD. A large, multi-centre randomised controlled trial (RCT) would influence and inform national guidance by

- Establishing if clinical markers of CD are improved by supplementation in patients with vitamin D deficiency
- Confirm if quality of life is affected by vitamin D supplementation in those deplete
- Establishing whether there is a need for routine screening for vitamin D deficiency in patients with CD
- Establishing if there is an effective vitamin D supplementation dose in patients with CD

To answer these questions with authoritative evidence of clinical and cost-effectiveness, a multi-centre randomised controlled trial (RCT) is required with a parallel economic evaluation. However, as per Medical Research Council guidelines [23] smaller feasibility studies should be conducted first to test methodology and establish parameters for a full RCT. This will help ensure success and appropriate use of resources in a definitive trial.

## Objectives

### Screening study

Screening was done to determine the prevalence of vitamin D deficiency in adults with CD in Birmingham, UK during autumn and winter measured by 25(OH) D testing.

### Feasibility trial

Trial was done to assess the feasibility of conducting a national, multi-site RCT in adult patients with CD and vitamin D deficiency and to determine whether vitamin D supplementation improves clinical markers, symptoms of disease and reported disease-related disability via assessments of:

- Patient identification and recruitment in to the feasibility trial
- Identification and analysis of any failure to recruit or retain participants
- Acceptability to patients of taking a daily oral vitamin supplement for 24 weeks
- Acceptability to patients of completing questionnaires and blood tests to be carried out during the study
- Trial processes, including biochemical and clinical measurements of Vitamin D metabolites and hepcidin blood levels and Crohn’s Disease Activity Index (CDAI)
- Safety of the study intervention and any adverse events

## Trial design

D-CODE is a two-part study entailing two vitamin D screening studies and an interventional feasibility study.

The vitamin D screening studies will take place during autumn and winter to determine the prevalence of vitamin D deficiency in patients with CD during autumn and winter.

In the intervention/feasibility stage, D-CODE is an exploratory study to assess the feasibility of an open-label, multi-site, superiority randomised controlled trial. Participants with vitamin D deficiency (< 50 nmol/L serum 25-hydroxyvitamin D [25(OH)D]) will be randomised to one of two parallel arms (A or B) with a primary endpoint of improvement in symptoms and health-related quality of life after 24 weeks of treatment for vitamin D deficiency in patients with CD. The control group (A) will receive a low dose of vitamin D3 oral capsule 400 IU daily for 24 weeks. The intervention group (B) will receive a higher treatment dose regimen of vitamin D3 oral capsule 3200 IU daily for 12 weeks followed by 800 IU daily for 12 weeks. This dose is well within the upper tolerable limit of 4000 IU daily set by the European Food Standards Agency [24]. Table 1 depicts the participant pathway and timeline. The D-CODE protocol was designed in accordance with the Standard Protocol Items: Recommendations for Interventional Trials (SPIRIT) statement [25]. The trial has been registered with ClinicalTrials.gov identifier NCT03718182 and ISRCTN number 15717783.

**Table 1:** Participant pathway and timeline and interventions

Procedures	Routine Crohn's out-patient appointment	Telephone contact 1 week	Feasibility trial visits					Telephone and postal
						Follow-up visit 1 12 weeks ± 7 days	Follow-up visit 2 24 weeks ± 14 days	Follow-up visit 3 28 weeks ± 14 days
	Vitamin D screening		Baseline	Eligibility	Randomisation	Intervention phase		Final follow-up
Informed consent	X		X					
Demographics	X							
Medical history including current medication list	X		X					
Confirmation of eligibility	X			X				
Vitamin D dried blood spot test	X							
Answer lifestyle and food frequency questions	X							X
Telephone contact to arrange baseline appointment for feasibility trial		X						
Randomisation					X			
*Pregnancy test as applicable*			X					
*Blood tests:*								
Vitamin D (25(OH)D)			X			X	X	
Calcium			X			X	X	
Parathyroid hormone			X			X	X	
Albumin			X			X	X	
C-reactive protein			X			X	X	
Iron studies			X			X	X	
Ferritin			X			X	X	
Full blood count			X			X	X	
Hepcidin			X			x	X	
Vitamin D metabolites			X			X	X	
Height			X					
Weight			X			X	X	
Stool sample for faecal calprotectin ^a^					x		x	
PROM 1 IBDQ					X		X	
PROM 2 EQ-5D-5L					X		X	
Clinical Assessment 3 CDAI					X	X	X	
Adverse event/reaction assessments						X	X	X
Dispensing of trial medication					X	X		
Review of treatment diary						X	X	
Compliance (pill count)						X	X	
Assessment 4 Closing participant experience questionnaire - postal								X

^a^If a faecal calprotectin sample has been taken by the participants of the clinical team within the previous month, a further sample is not required for the study. The clinical result can be used.

## Methods: participants, interventions and outcomes

### Study setting

The D-CODE study is set in secondary and tertiary care centres in Birmingham, UK. Birmingham is a multi-cultural and ethnically diverse city. Centre selection includes one large tertiary referral centre and one large and one smaller secondary care centre for patients with CD. The three participating sites have been chosen to give an effective cross section of different demographic and ethnic groups across the city.

### Eligibility criteria

Inclusion criteria for screening studies are as follow:

1. Those with a confirmed diagnosis of CD
2. Greater than or equal to 18 years of age
3. Have provided written informed consent

Inclusion criteria for the feasibility trial are as follow:

1. Those with a confirmed diagnosis of CD
2. Identified as having vitamin D deficiency < 50 nmol/L 25(OH)D in the screening study
3. Greater than or equal to 18 years of age
4. Already receiving treatment for CD as per NICE Guidance or those in remission and not currently receiving treatment but who continue to attend out-patient appointments in hospital
5. Has provided written informed consent

Exclusion criteria for the feasibility trial are as follow:

1. Currently taking over the counter vitamin D, fish oil or multivitamin supplementation and unwilling to stop this to participate in the feasibility trial
2. Currently receiving vitamin D-containing supplementation prescribed by a healthcare professional
3. Currently receiving bisphosphonates
4. Currently receiving Digitalis or other cardiac glycosides
5. Currently receiving phenytoin
6. Currently receiving barbiturates (e.g., Amylobarbitone, Butobarbitone, Methyl Phenobarbitone, Pentobarbitone, Quinalbarbitone,)
7. Currently receiving actinomycin
8. Currently receiving imidazole
9. With known hyperparathyroidism
10. With known sarcoidosis
11. With known renal disease or kidney stones
12. With known hypercalcaemia (corrected calcium ≥ 2.60 mmol/L)
13. With known underlying liver disease
14. With known hypersensitivity to vitamin D supplements or any of the trial medication excipients
15. Who are pregnant, breast feeding, trying to conceive or women of child-bearing capacity who decline to have a pregnancy test where applicable and/or decline to take effective contraceptive measures during the intervention period
16. Individuals who have participated in a trial testing a medicinal product within 6 months preceding screening

Patients taking iron supplementation are not excluded as iron is a common treatment used within CD management.

### Who will take informed consent?

Informed written consent will be taken from participants prospectively by the site study team. Only adults (> 18 years old) who have the capacity to provide consent to participate will be included. Prospective consent will be collected separately for each part of the study and consent re-confirmed at each study visit. A patient information leaflet is provided for each discrete part. This is because the majority of participants who take part in the vitamin D screening study will not be recruited into the intervention feasibility trial. The study will be conducted according to the principles of Good Clinical Practice (GCP) and to the ethical principles that have their origin in the Declaration of Helsinki.

### Additional consent provisions for collection and use of participant data and biological specimens

Not applicable

### Interventions

#### Explanation for the choice of comparators

- Vitamin D3 is recommended by the National Osteoporosis Society [16] as being the most effective form of vitamin D supplementation.
- Cholecalciferol is a licensed product in the given doses and is commercially available.

#### *Arm A: * Cholecalciferol 400 IU daily

The rationale is as follows:

- Although some studies have suggested that a dose of 400 IU is too low to be effective [26], it remains the standard dose recommended by NICE [27] for treatment of ‘at risk’ groups.

#### *Arm B:* Cholecalciferol 3200 IU daily for 12 weeks followed by Cholecalciferol 800 IU daily for a further 12 weeks

The rationales are as follow:

- The National Osteoporosis Society recommend a period of a loading dose followed by a period of maintenance dose [16].
- The European Food Safety Authority [24] set an upper tolerable limit of 4000 IU daily in adults. 4000 IU is the dose recommended by the Endocrine Society to treat vitamin D deficiency in those over 8 years old [28].
- It is feasible that a higher dose regimen of vitamin D will offer greater clinical benefits in patients with CD in terms of effectively treating their vitamin D deficiency.

#### Treatment period

The total treatment period is 24 weeks for both groups. This is an adequate amount of time for vitamin D levels to increase to normal limits [16] and for any changes in patient-reported outcomes to be recognised.

##### Intervention description

In *Arm A*, Cholecalciferol 400 IU capsules are to be taken orally once daily for a total of 24 weeks.

In *Arm B*, Cholecalciferol 3200 IU capsules are to be taken orally once daily for 12 weeks followed by Cholecalciferol 800 IU capsules to be taken once daily for 12 weeks.

The capsules have a UK marketing authorisation. They are being used within the terms of the marketing authorisation and have not been repackaged for use in the trial. Therefore, an Annexe 13 label is not required, and a normal pharmacy dispensing label will be used in accordance with Schedule 5 of The Medicines for Human Use (Marketing Authorisations Etc.) Regulations 1994.

Participants will return unused capsules at the 12 and 24 weeks follow-up appointments.

#### Criteria for discontinuing or modifying allocated interventions

Patients may prematurely discontinue treatment for any of the following reasons:

- The patient withdraws consent.
- Unacceptable toxicity which requires the treatment to be permanently discontinued
- Inter-current illness preventing further treatment.
- Any change in the patient’s condition that justifies the discontinuation of treatment in the clinician’s opinion.
- A participant becomes pregnant during the trial.

For the follow-up, as per Table 1, participant timeline will continue unless the patient explicitly also withdraws consent for follow-up.

#### Strategies to improve adherence to interventions

Participants will be asked to return all unused vitamin D supplements to the dispensing pharmacy. Participants will also be given a treatment diary to record each day that they take their vitamin D capsule. The treatment diary will be reviewed at the follow-up appointments at weeks 12 and 24 as an indication of compliance. Participants will also give a pragmatic indication of compliance by indicating if they think they have taken all, most, some or none of their capsules.

#### Relevant concomitant care permitted or prohibited during the trial

As per the product SmPC, concomitant treatment with Phenytoin or barbiturates can decrease the effect of vitamin D because of metabolic activation. Concomitant use of glucocorticoids can decrease the effect of vitamin D. Simultaneous treatment with ion exchange resins such as cholestyramine/colesevelam or laxatives such as paraffin oil may reduce the gastrointestinal absorption of vitamin D. The cytotoxic agent actinomycin and imidazole antifungal agents interfere with vitamin D activity by inhibiting the conversion of 25-hydroxyvitamin D to 1,25-dihydroxyvitamin D by the kidney enzyme, 25-hydroxyvitamin D-1-hydroxylase.

The effects of digitalis and other cardiac glycosides may be accentuated with the oral administration of calcium combined with Vitamin D.

However, some of these medications are common treatments in patients with CD such as glucocorticosteroids and cholestyramine/colesevelam. Therefore, these will be allowed in the study as usual adjunct therapies in the management of CD; as some of these medications may act to decrease effectiveness of vitamin D, and there is no increased risk of toxicity for these participants.

The following concomitant medications are prohibited within the study:

- Vitamin D, fish oil or multivitamin supplementation
- Bisphosphonates
- Digitalis or other cardiac glycosides
- Phenytoin
- Barbiturates (e.g., amylobarbitone, butobarbitone, methyl phenobarbitone, pentobarbitone, quinalbarbitone)
- Actinomycin
- Imidazole

Participant’s medication history will be recorded at baseline and reviewed by a delegated doctor when confirming eligibility prior to randomisation, including any medications that have been started or stopped within the 2 weeks prior to randomisation. Medication history will then be checked again at 12- and 24-week visits. All medications not listed in the prohibited concomitant medication list are permitted within both treatment arms.

#### Provisions for post-trial care

Where participants have perceived benefit from the vitamin D supplementation, a letter will be sent to their GP asking them to review this with the patient and consider continued use.

#### Outcomes

In screening studies, 25(OH) vitamin D results are measured in participants to determine prevalence of vitamin D deficiency < 50 nmol/L during autumn and winter.

In the feasibility trial, outcomes to assess are the following:

1. Consent rate
2. Compliance rate
3. Retention rate
4. Completion rates of efficacy outcomes
5. Adverse events

The feasibility of implementing the following measures in the definitive trial will be assessed:

Primary outcome: Inflammatory Bowel Disease Questionnaire (IBDQ)

Secondary outcomes:

1. EQ-5D-5L
2. Crohn’s Disease Activity Index Score (CDAI)
3. Vitamin D (25(OH)D)
4. Vitamin D metabolites
5. Corrected calcium
6. Parathyroid hormone
7. Hepcidin
8. Iron deficiency anaemia
9. Inflammation
10. Faecal calprotectin

### Participant timeline

Table 1 shows the participant timeline and schedule of interventions in accordance with SPIRIT.

### Sample size

As this is a feasibility study, a formal sample size calculation is not appropriate; the study is not powered to detect a clinically important difference in any outcomes between the two treatment groups. Instead, the aims are to provide robust estimates of the likely rates of recruitment, consent and follow-up and to gain estimates of the outcome event rates to accurately inform power calculations for a future definitive trial. Various sample sizes ranging from 24 to 50 have been recommended for feasibility studies [29–31]. A sample size of 50, the upper range, has been selected to allow the aims of the study to be addressed with more confidence.

Fifty patients will be recruited to the intervention part of the study. Allowing for 20% loss to follow-up, data will be available for 40 patients. In order to recruit 50 patients into the feasibility trial, 250 patients will need to be screened to allow for the following:

- Fifty percent of screened patients having vitamin D deficiency
- Twenty percent of patients with vitamin D deficiency not eligible
- Fifty percent of eligible patients not consenting

If we identify 100 eligible patients, we will be able to estimate a consent rate of 50% to within a confidence interval of ± 10%. If 50 patients are recruited, we will be able to estimate a retention rate and completion rate (of efficacy outcomes) of 80% to within a confidence interval of ± 11%. If we obtain a complete data on 40 patients, we will be able to estimate the compliance rate and proportion of patients not experiencing an adverse event (AE) of 80% to within a confidence interval of ± 12%.

The target sample size is divided between the three hospital settings to ensure a mix of patients, according to the size of the hospital and likely patient numbers. For each of the two larger hospitals, in the screening study, the target is 100 participants, with 20 participants for the intervention study. The smaller hospital target is 50 participants in the screening study and 10 participants in the intervention study.

### Recruitment

A local retrospective audit identified that over a 2-month period (March–April 2015) at the Queen Elizabeth Hospital Birmingham, a site of University Hospitals Birmingham NHS Foundation Trust (UHBFT), 323 patients with CD were booked into Gastroenterology out-patient clinics [32]. It is therefore reasonable to assume that sufficient patients will attend Gastroenterology clinics across three sites of UHBFT over a 3-month period.

#### Participant identification—screening study

Patients attending for their routine CD follow-up out-patient appointment will be identified by their Gastroenterologist for possible inclusion in the screening study. The Gastroenterologist seeing the patient will identify if, in their professional opinion, the patient has a confirmed diagnosis of CD (informed by clinical history, examination, imaging, endoscopy and/or biopsy results) and discuss the screening study with the patient avoiding any coercion. If the patient is interested in participating, the Gastroenterologist will direct the patient to the study team within the Gastroenterology Clinic for consent and participation.

#### Participant identification—intervention study

Participants from the screening study with a vitamin D result < 50 nmol/L and who meet the rest of the eligibility criteria from their reported medical history, may be identified for possible inclusion in the feasibility trial within the recruitment period. The study team will screen potentially eligible patients and contact them by telephone to explain the intervention study and invite them to a baseline appointment if they are interested in participating.

### Assignment of interventions: allocation

#### Sequence generation

Randomisation lists will be generated using block randomisation with random variable block length, stratified by site. The lists will be produced by an independent statistician (who is not otherwise involved in the D-CODE feasibility trial) at the Liverpool Clinical Trials Centre (LCTC).

#### Implementation

Participants will be randomised (in a ratio of 1:1) to low-dose vitamin D (400 IU) or treatment dose vitamin D (3200 IU/800 IU) using a secure (24-h) web-based randomisation programme.

#### Concealment mechanism

The randomisation programme is controlled centrally by Liverpool Clinical Trials Centre (LCTC), University of Liverpool to ensure allocation concealment.

### Assignment of interventions: blinding

#### Who will be blinded

D-CODE is an open-label study; therefore, blinding will not be implemented.

#### Procedure for unblinding if needed

D-CODE is an open-label study; therefore, blinding will not be implemented.

### Data collection and management

#### Plans for assessment and collection of outcomes

**Screening study**

Data will be collected from participants regarding modifiable risk factors for vitamin D deficiency including use of over-the-counter or prescribed vitamin D-containing supplementation, sun exposure, diet and smoking. Participants will be asked questions related to

- Usual skin exposure to the sun. This will be assessed by the participants reported wearing usual clothing worn during warm/sunny weather
- Use of sun protection factor creams/lotion
- Travel within the previous 3 months to a warm/sunny country outside of the UK

Participants will be asked food frequency questions concerning weekly consumption of key vitamin D-containing foods including oily fish, red meat, liver, eggs with the yolk and other fortified foods. Frequency will be determined as rarely/never, 1–2, 3–4 or > 5 times per week.

Use of vitamin D-containing supplementation will be a ‘yes or no’ to over-the-counter or prescribed supplements and smoking will be a ‘yes-or-no’ response.

**Intervention study**

***Quality of life and patient experience***

Patient-reported outcomes have been included to measure perceived improvement in symptoms and disease related quality of life for participants.

The Inflammatory Bowel Disease Questionnaire (IBDQ )[33], a disease-specific questionnaire for adult patients with IBD, will be used to measure disease-related quality of life. In a systematic review of patient-reported outcomes in IBD, Chen et al. [34] reported that the IBDQ has good validity, reliability and internal consistency, and excellent content validity and cross-cultural validity; based on the COSMIN checklist 4-point scale rating of patient reported outcomes. The EQ-5D-5L (Euroqol) [35], a generic health utility measure, will also be used to help identify unanticipated effects of treatment not captured in a disease-specific questionnaire. The EQ-5D-5L has been shown to have good validity, reliability and responsiveness in an evaluation of 502 patients with IBD (CD *n* = 270, UC *n* = 232) [36]. The two questionnaires (IBDQ and EQ-5D-5L) will be presented as a single, printed paper-based booklet at the randomisation appointment and after 24 weeks (end of intervention). The endpoint will be an improvement from baseline to 24 weeks in the participants perceived health-related quality of life.

In addition, The Crohn’s Disease Activity Index (CDAI) [37] will be calculated at 0, 12 and 24 weeks to quantify the symptoms of patients with CD. The CDAI is a widely used research and clinical tool that has been extensively validated. The web-based CDAI-Calculator from IBD support as recommended by the European Crohn’s Colitis Organisation will be used to calculate the CDAI score from non-identifiable patient data (https://www.ibdsupport.org.au/cdai-calculator).

A short-closing questionnaire will be posted to participants prior to their final telephone follow-up with a pre-paid return envelope at 28 weeks. This will be a two-point questionnaire to explore participants’ experience and views towards being involved in research.

#### Plans to promote participant retention and complete follow-up

If voluntary withdrawal occurs, the patient should be asked to allow continuation of scheduled evaluations and complete an end-of-study evaluation if appropriate. In the event of an AE, the patient should be asked to allow appropriate care under medical supervision until the symptoms of any adverse event resolve or the patient’s condition becomes stable. Follow-up of these patients will be continued through the PI at each centre and, where these are unsuccessful, through the patient’s GP, unless the participant explicitly also withdraws consent for follow-up.

Where patients are thought to be lost to follow-up, attempts will be made to contact the patient: (a) in writing via the address provided or (b) follow-up telephone call via the telephone number provided at recruitment. Where it is thought that patient's contact details may have changed, the following sources will be checked for up-to-date contact details: (a) contact details currently held by the managing hospital site and (b) contact details currently held by the patient’s GP.

### Data management

The D-CODE paper case report form (CRF) is the primary data collection instrument for the study. Completed CRFs will be sent to the LCTC. Patient questionnaires and diaries are a source document. These will be photocopied with a copy retained at site and originals sent to LCTC. Data will be collected pseudo-anonymised with only participants’ randomisation number and date of birth recorded on the data collection tools.

#### Confidentiality

Individual participant medical information obtained as a result of this study is considered confidential, and disclosure to third parties is prohibited with the exceptions noted below.

CRFS will be labelled with the patient’s unique trial screening and/or randomisation number. Medical information may be given to the participant’s medical team and all appropriate medical personnel responsible for the participant’s welfare. The LCTC will not be undertaking activities requiring the transfer of identifiable data.

#### Plans for collection, laboratory evaluation and storage of biological specimens for genetic or molecular analysis in this trial/future use

**Screening study**

Vitamin D dried blood spot tests will be used for screening patients for vitamin D deficiency in the screening study. This blood test is taken using the finger prick sample collection kit which is CE marked and supplied by City Assays, Sandwell and West Birmingham NHS Trust (http://www.cityassays.org.uk) and analysed by the Sandwell and West Birmingham Laboratory; Patients and their GP will be informed of the vitamin D result from the screening study by post, whether they participate in the intervention study or not.

**Intervention study**

***Biological specimens for assessing safety***

Blood samples will be taken and biochemical measures will be used for assessing safety and efficacy during the trial. Safety measures include serum vitamin D 25(OH)D, corrected calcium and parathyroid hormone (PTH).

Further biochemical measures will be carried out as follows to inform study results:

- Inflammation: CRP will be measured to determine the presence or absence of inflammation. Faecal calprotectin will be measured as an indication of CD activity and active inflammation. In clinical practice, this is an accepted method for monitoring inflammation in CD and is less invasive than endoscopic methods.
- Iron deficiency anaemia: Full blood count and iron studies (serum iron, total iron binding capacity, iron saturations, transferrin and ferritin) will be performed to determine the presence or absence of iron deficiency anaemia.

Hepcidin will be measured for exploratory and not diagnostic purposes. A standard serum separation tube will be used to collect samples. In the laboratory, samples will be centrifuged within 1 day and serum stored at − 80 °C until analysis at Birmingham Heartlands Hospital. This is not a routine assay and will be analysed as a batch at the end of study to ensure consistency.

Serum vitamin D status is conventionally assessed through measurement of serum levels of 25(OH)D, the major circulating form of vitamin D. However, it is now clear that many other vitamin D metabolites are involved in biological responses to vitamin D, notably the active, hormonal, form 1,25-dihydroxyvitamin D (1,25(OH)2D). Researchers at the University of Birmingham have developed new methodologies to measure multiple metabolites of vitamin D, alongside 25(OH)D, in single serum samples [38]. These methods will be used to provide a more detailed perspective of vitamin D status in patients with Crohn’s disease and determine if metabolites other than 25(OH)D are linked to changes in the disease.

Samples will be collected in an EDTA sample tube. In the laboratory, samples will be centrifuged within 1 day and serum stored at − 20 °C until analysis at the Institute of Metabolism and Systems Research, University of Birmingham. Samples will be analysed in batches to ensure consistency.

### Statistical methods

#### Statistical methods for primary and secondary outcomes

Screening data will be presented numerically, along with reasons why patients are ineligible, unwilling to provide consent or are consented but not randomised. Baseline data will be presented descriptively to ensure that the patients that have been recruited are representative of the target population. As this is a feasibility study and consequently not powered to detect a significant difference between groups in the main outcome measures, no comparative analyses are planned. The main outcome measures will be presented using summary statistics, and the proportion of missing values will be assessed. The number of withdrawals (and reasons) will be presented in each arm. Adverse events will also be presented split by treatment arm.

### Interim analyses

There will be no formal interim analysis but accumulating data will be presented at regular intervals (at least annually) for review by an Independent Data Monitoring and Safety Committee (IDSMC). These analyses will be performed at the LCTC. The IDSMC will be asked to give advice on whether the accumulated data from the trial, together with results from other relevant trials, justifies continuing recruitment of further patients or further follow-up. A decision to discontinue recruitment, in all patients or in selected subgroups will be made only if the result is likely to convince a broad range of clinicians including participants in the trial and the general clinical community.

### Methods for additional analyses (e.g., subgroup analyses)

Not applicable

### Methods in analysis to handle protocol non-adherence and any statistical methods to handle missing data

Not applicable

### Plans to give access to the full protocol, participant level-data and statistical code

Not applicable

### Oversight and monitoring

#### Composition of the coordinating centre and trial steering committee

A Trial Management Group (TMG) will be formed comprising the Chief Investigator, other lead investigators (clinical and nonclinical) and members of the LCTC. The TMG will be responsible for the day-to-day running and management of the trial.

The Trial Steering Committee (TSC) will consist of an independent chairperson, 2 independent experts in the field of Gastroenterology or Endocrinology and a biostatistician, patient and public representation and up to seven Principal Investigators. The role of the TSC is to provide overall supervision for the trial and provide advice through its independent Chairman. The ultimate decision for the continuation of the trial lies with the TSC.

#### Composition of the data monitoring committee, its role and reporting structure

The Independent Data and Safety Monitoring Committee (IDSMC) consists of an independent chairperson plus 2 independent members: one of whom is an expert in the field of IBD, and one who is an expert in medical statistics. The IDSMC will be responsible for reviewing and assessing recruitment, interim monitoring of safety and effectiveness, trial conduct and external data. The IDSMC will first convene prior to the start of recruitment and will then define frequency of subsequent meetings (at least annually). The IDSMC will provide a recommendation to the Trial Steering Committee concerning the continuation of the study.

#### Adverse event reporting and harms

Safety reporting of adverse events or reactions will be actively monitored during the feasibility trial from the period of randomisation until the 28th-week follow-up appointment. For the purposes of the D-CODE feasibility trial the following AEs will be

*Included*

- An exacerbation of a pre-existing illness (excluding CD)
- An increase in frequency or intensity of a pre-existing episodic event/condition (excluding CD)
- A condition (even though it may have been present prior to the start of the trial) detected after trial medication administration
- Laboratory abnormalities that require clinical intervention or further investigation (unless they are associated with an already reported clinical event).
- Abnormalities in physiological testing or physical examination that require further investigation or clinical intervention
- Injury or accidents
- Symptomatic overdose of trial medication
- Vitamin D toxicity or hypercalcaemia (both symptomatic and asymptomatic)

*Excluded*

- Medical or surgical procedures—the condition which leads to the procedure is the adverse event
- Pre-existing disease or conditions present before treatment that do not worsen
- Situations where an untoward medical occurrence has occurred (e.g., cosmetic elective surgery)
- The disease being treated or associated symptoms/signs, unless more severe than expected for the patient’s condition

A suspected unexpected serious adverse reaction (SUSAR) is an adverse reaction that is classed in nature as serious and which is not consistent with the information about the IMP in question, which in the case of a licensed product is set out in the SPC for that product.

A serious adverse event is defined as an adverse event that

- results in death;
- is life threatening;
- requires hospitalisation or prolongation of existing hospitalisation;
- results in persistent or significant disability or incapacity;
- consists of a congenital anomaly or birth defect;
- other important medical events.

Serious adverse events will require expedited reporting.

#### Notification of deaths

Death is not an expected outcome for participants in the study, either from the intervention or from the participants underlying CD. Therefore, participant death during the study will be classed as a SUSAR and will require expedited reporting.

#### Frequency and plans for auditing trial conduct

The LCTC performs regular internal auditing of LCTC studies and LCTC systems and processes. In addition, the study Sponsor conducts a programme of audit carried out by the Trusts Research and Development Governance department.

#### Plans for communicating important protocol amendments to relevant parties (e.g., trial participants, ethical committees)

Regulatory approval will be sought for all amendments to the protocol and communicated to sites and affected participants.

### Dissemination plans

Results will be disseminated via publication and presentation at clinical and scientific conferences. In addition, the trial website will be updated in a timely manner to ensure progress reports, and results are easily accessible to a wide audience. Results will be disseminated regardless of the magnitude or direction of effect.

Key target audiences are nurses and medics working in gastroenterology areas. These practitioners are key in influencing changes in everyday practice in terms of screening for vitamin D deficiency in patients with CD.

## Discussion

The aim of this study is to determine if it is feasible to carry out D-CODE as a full multi-centre RCT. There are few RCTs investigating the general non-skeletal effects of vitamin D supplementation [39]. Only about a quarter of these include participants with an identified vitamin D deficiency, and in terms of CD, evidence for vitamin D supplementation is mostly observational with some RCTs giving conflicting evidence [39, 40]. It is reasonable to assume that patients who are deficient in vitamin D will gain the greatest benefit from supplementation. A key aspect of D-CODE is the identification of vitamin D deficiency prior to supplementation.

Determining a cutoff point for deficiency is contentious with the Scientific Advisory Committee on Nutrition (SACN) [41] in the UK determining vitamin D deficiency as levels < 25 nmol/L 25(OH)D in terms of population risk of bone diseases. However, most professional bodies recognise that this cutoff is too low and recommend vitamin D levels of at least 50 nmol/L [16, 28], with some suggestion that 75 nmol/L is beneficial in those with diseases such as CD [42, 43]. In D-CODE, we have opted for the more conventional cutoff of 50 nmol/L.

Although vitamin D supplementation is available as a combined therapy with calcium, a randomised, placebo-controlled trial found that participant compliance was better with vitamin D supplementation alone as added calcium may cause gastrointestinal side effects [44].

The primary outcome measure of the main RCT will be the Inflammatory Bowel Disease Questionnaire (IBDQ). Patient-reported outcomes are becoming increasingly important in research. Where biochemical measures may give an indication of disease activity and efficacy of treatment, the interpretation of this into lived benefit for the patient is essential. Hence, two questionnaires are being used in D-CODE to collect disease-specific and generic health-related quality of life data. The measurement of vitamin D metabolites beyond the usual and the measurement of hepcidin in the study offer opportunities to explore novel aspects of how vitamin D deficiency and supplementation may impact on CD activity and the association with iron deficiency anaemia in this group of patients.

Blinding is often an important aspect of randomised clinical trials [45]. Therefore, the unblinded nature of this study is recognised as a limitation. In the feasibility stage, no comparative analysis is planned. Estimates of the outcome event rates will be assessed; therefore, we do not anticipate that the unblinded nature of the feasibility study will adversely affect results. In a definitive trial blinding will be desirable to prevent bias.

Ultimately, it is hoped that this feasibility study will lead to a definitive trial that will investigate the benefits of treating vitamin D deficiency in patients with CD. This valuable evidence may then be used to inform national guidance and ensure parity in clinical practice.

## Trial status

We are still in the process of recruiting.

## Abbreviations

AE: Adverse event
AR: Adverse reaction
CD: Crohns disease
CDAI: Crohns Disease Activity Index
CRF: Case report form
CTIMP: Clinical Trials of an Investigational Medicinal Product
CTU: Clinical Trials Unit
EUDRACT: European Clinical Trials Database
GCP: Good clinical practice
GP: General practitioner
HRA: Health Research Authority
IBD: Inflammatory bowel disease
IBDQ: Inflammatory Bowel Disease Questionnaire
IDSMC: Independent Data and Safety Monitoring Committee
LCTC: Liverpool Clinical Trials Centre
NICE: National Institute for Health and Care Excellence
RCT: Randomised controlled trial
SACN: Scientific Advisory Committee on Nutrition
TMG: Trial Management Group
TSC: Trial Steering Committee
UHBFT: University Hospitals Birmingham NHS Foundation Trust
UK: United Kingdom
25(OH)D: 25-hydroxyvitamin D

## References

[1] Crohn's and Colitis UK, editor. Crohn's disease: your guide. UK: Crohn's and Colitis; 2019. p. 9. https://crohnsandcolitis.org.uk/about-crohns-and-colitis/publications/crohns-disease.

[2] Casellas F (2001). Influence of inflammatory bowel disease on different dimensions of quality of life. Eur J Gastroenterol Hepatol.

[3] Gomollón F (2016). 3rd European evidence-based consensus on the diagnosis and management of Crohn’s disease 2016: part 1: diagnosis and medical management. J Crohns Colitis.

[4] Chatu S (2013). Factors associated with vitamin D deficiency in a multicultural inflammatory bowel disease cohort. Frontline Gastroenterol.

[5] Caviezel D (2017). High prevalence of vitamin D deficiency among patients with inflammatory bowel disease. Inflamm Intestinal Dis.

[6] Sadeghian M (2016). Vitamin D status in relation to Crohn's disease: meta-analysis of observational studies. Nutrition.

[7] Mouli VP, Ananthakrishnan AN (2014). Review article: vitamin D and inflammatory bowel diseases. Aliment Pharmacol Ther.

[8] Scott FI (2016). Risk of nonmelanoma skin cancer associated with the use of immunosuppressant and biologic agents in patients with a history of autoimmune disease and nonmelanoma skin cancer. JAMA Dermatol.

[9] Vitek L (2015). Bile acid malabsorption in inflammatory bowel disease. Inflamm Bowel Dis.

[10] Smith EM, Tangpricha V (2015). Vitamin D and anemia: insights into an emerging association. Curr Opin Endocrinol Diabetes Obes.

[11] Sim JJ (2010). Vitamin D deficiency and anemia: a cross-sectional study. Ann Hematol.

[12] Patel NM (2010). Vitamin D deficiency and anemia in early chronic kidney disease. Kidney Int.

[13] Bacchetta J (2014). Suppression of iron-regulatory hepcidin by vitamin d. J Am Soc Nephrol.

[14] Nemeth E, Ganz T (2009). The role of hepcidin in iron metabolism. Acta Haematol.

[15] Basseri RJ, et al. Hepcidin is a key mediator of anemia of inflammation in Crohn's disease. J Crohns Colitis. 2012;7(8):e286–91. 10.1016/j.crohns.2012.10.013 23219355

[16] Francis RM (2018). National Osteoporosis Society: Vitamin D and bone health: a practical clinical guideline on patient management. Maturitas.

[17] National Institute for Health and Care Excellence, Crohn's disease (2019). Management (NG129).

[18] Lewis N, Scott B. Guidelines for osteoporosis in inflammatory bowel disease and coeliac disease. Bri Soc Gastroenterol. 2007.

[19] Ananthakrishnan AN (2013). Normalization of plasma 25-hydroxy vitamin D is associated with reduced risk of surgery in Crohn's disease. Inflamm Bowel Dis.

[20] Stio M (2004). Effect of anti-TNF therapy and vitamin D derivatives on the proliferation of peripheral blood mononuclear cells in Crohn's disease. Digest Dis Sci.

[21] Reich KM (2014). Vitamin D improves inflammatory bowel disease outcomes: basic science and clinical review. World J Gastroenterol.

[22] Hlavaty T, Krajcovicova A, Payer J (2015). Vitamin D therapy in inflammatory bowel diseases: who, in what form, and how much?. J Crohns Colitis.

[23] Craig P (2008). Developing and evaluating complex interventions: the new Medical Research Council guidance. BMJ.

[24] Efsa Panel on Dietetic Products, N. and Allergies (2012). Scientific opinion on the tolerable upper intake level of vitamin D. EFSA Journal.

[25] Chan AW (2013). SPIRIT 2013 statement: defining standard protocol items for clinical trials. Ann Intern Med.

[26] Suibhne TN (2012). Vitamin D deficiency in Crohn's disease: prevalence, risk factors and supplement use in an outpatient setting. J Crohns Colitis.

[27] National Institute for Health and Care Excellence, Vitamin D (2017). Supplement use in specific population groups PH56.

[28] Holick MF (2011). Evaluation, treatment, and prevention of vitamin D deficiency: an Endocrine Society clinical practice guideline. J Clin Endocrinol Metab.

[29] Lancaster GA, Dodd S, Williamson PR (2004). Design and analysis of pilot studies: recommendations for good practice. J Eval Clin Pract.

[30] Julious SA (2005). Sample size of 12 per group rule of thumb for a pilot study. Pharm Stat.

[31] Sim J, Lewis M (2012). The size of a pilot study for a clinical trial should be calculated in relation to considerations of precision and efficiency. J Clin Epidemiol.

[32] Fletcher J, Swift A (2017). Vitamin D screening in patients with inflammatory bowel disease. Gastrointestinal Nursing.

[33] Irvine EJ (1999). Development and subsequent refinement of the inflammatory bowel disease questionnaire: a quality-of-life instrument for adult patients with inflammatory bowel disease. J Pediatr Gastroenterol Nutr.

[34] Chen X-L (2017). Inflammatory bowel disease-specific health-related quality of life instruments: a systematic review of measurement properties. Health Qual Life Outcom.

[35] Herdman M (2011). Development and preliminary testing of the new five-level version of EQ-5D (EQ-5D-5L). Qual Life Res.

[36] Stark RG (2010). Validity, reliability, and responsiveness of the EQ-5D in inflammatory bowel disease in Germany. Inflamm Bowel Dis.

[37] Best WR (1976). Development of a Crohn's disease activity index. National Cooperative Crohn's Disease Study. Gastroenterology.

[38] Jenkinson C (2018). Analysis of multiple vitamin D metabolites by ultra-performance supercritical fluid chromatography-tandem mass spectrometry (UPSFC-MS/MS). J Chromatogr B Analyt Technol Biomed Life Sci.

[39] Rejnmark L (2017). Non-skeletal health effects of vitamin D supplementation: a systematic review on findings from meta-analyses summarizing trial data. PLOS ONE.

[40] Scragg R (2018). Limitations of vitamin D supplementation trials: why observational studies will continue to help determine the role of vitamin D in health. J Steroid Biochem Mol Biol.

[41] Scientific Advisory Committee on Nutrition (2016). SACN vitamin D and health report.

[42] Nielsen OH, Rejnmark L, Moss AC (2018). Role of vitamin D in the natural history of inflammatory bowel disease. J Crohns Colitis.

[43] Nielsen OH, et al. Managing vitamin D deficiency in inflammatory bowel disease. Frontline Gastroenterol. 2019:10(4):394–400. 10.1136/flgastro-2018-101055 PMC6788352 31656565

[44] Grant AM (2005). Oral vitamin D3 and calcium for secondary prevention of low-trauma fractures in elderly people (Randomised Evaluation of Calcium Or vitamin D, RECORD): a randomised placebo-controlled trial. Lancet.

[45] Karanicolas PJ, Farrokhyar F, Bhandari M (2010). Practical tips for surgical research: blinding: who, what, when, why, how? Canadian journal of surgery. J Can Chirurgie.

